# Perioperative assessment of electroencephalography in dogs with congenital portosystemic shunts

**DOI:** 10.1093/jvimsj/aalaf051

**Published:** 2026-01-21

**Authors:** Adrien M Dupanloup, Peter J Dickinson, Christine M Toedebusch, Chelsea M Unkel, William T N Culp, Marguerite F Knipe

**Affiliations:** Department of Surgical and Radiological Sciences, School of Veterinary Medicine, University of California, Davis, CA 95616, United States; Department of Surgical and Radiological Sciences, School of Veterinary Medicine, University of California, Davis, CA 95616, United States; Department of Surgical and Radiological Sciences, School of Veterinary Medicine, University of California, Davis, CA 95616, United States; Department of Molecular Biosciences, School of Veterinary Medicine, University of California, Davis, CA 95616, United States; Department of Surgical and Radiological Sciences, School of Veterinary Medicine, University of California, Davis, CA 95616, United States; Department of Surgical and Radiological Sciences, School of Veterinary Medicine, University of California, Davis, CA 95616, United States

**Keywords:** electroencephalography, encephalopathy, hepatic, dog

## Abstract

**Background:**

Biomarkers to identify dogs at risk of developing seizures after surgical attenuation of congenital portosystemic shunts (CPS) have not been defined.

**Hypothesis/Objectives:**

To prospectively characterize perioperative electroencephalographic findings in dogs with CPS undergoing shunt attenuation, and to evaluate their association with outcomes after attenuation.

**Animals:**

Twenty-eight client-owned dogs with CPS enrolled prospectively.

**Methods:**

A prospective cohort of dogs presenting with no overt signs of encephalopathy underwent ambulatory electroencephalography (EEG) before and after CPS attenuation. Electroencephalography background activity and presence of epileptiform or encephalopathic features were assessed qualitatively. Quantitative analysis evaluated the mean dominant frequency and relative power of EEG frequency bands.

**Results:**

Dogs with a normal pre-attenuation EEG (*n* = 24) did not develop early-onset (<7 days) PAS; however, 8% (2/24) experienced late-onset seizures (>30 days post-attenuation). Four dogs had abnormal pre-attenuation EEG. Two of these dogs developed early-onset seizures and 3 dogs (75%) died before discharge due to worsening neurological signs (*n* = 2) or immediate postoperative complication (*n* = 1). Pre-attenuation covert encephalopathy defined by EEG showed a combination of epileptiform features (4/4), abnormal background rhythm (3/4), and a lack of graphoelements of sleep (2/4). Median ammonia concentration (range) was 102.5 μg/dL (34-401) in non-encephalopathic dogs and 145.5 μg/dL (56-275) in dogs with covert encephalopathy.

**Conclusions and clinical importance:**

Electroencephalography could provide useful diagnostic biomarkers to identify dogs at high risk of developing neurological complications after attenuation of CPS. In this cohort, dogs with no abnormalities detected on preoperative EEG had favorable outcomes during their hospitalization.

## Introduction

Dogs with congenital portosystemic shunts (CPS) often demonstrate varying degrees of hepatic encephalopathy (HE).^1^ Hepatic encephalopathy refers to a constellation of signs of neurological dysfunction, including but not limited to altered states of consciousness, compulsive behaviors, and seizures, that can occur in isolation or combination secondary to acute or chronic hepatopathy. These signs may episodically worsen due to precipitating factors such as ingestion of a high-protein meal or surgery. Various attenuation techniques have been developed to treat CPS, with the goal of diverting blood from the portal tributaries to the liver to allow for appropriate metabolism.^2^ While attenuation of CPS results in favorable outcomes in the majority of cases, some dogs may develop seizures after attenuation,^3^ referred to as post-attenuation seizures (PAS). The clinical course of PAS is heterogenous and may be refractory to anti-epileptic treatment.^3^^,^^4^ Dogs may additionally develop post-attenuation progressive encephalopathic signs such as worsening mentation, ranging from obtunded to comatose, which may ultimately result in death or euthanasia.^5–7^

Dogs with encephalopathic signs at time of CPS attenuation are at greater risk for developing PAS.^8^ However, identification of mild or subclinical HE is challenging in the clinical assessment of dogs with CPS. In human hepatology, the term “covert” or “minimal” HE refers to changes in electroencephalogram in the absence of overt HE or alternative diagnoses for neuropsychological impairment.^9^^,^^10^ Electroencephalography (EEG) has been used to diagnose and grade cortical dysfunction secondary to HE in humans^11^ where covert HE may predict the development of overt HE.^12^^,^^13^ There is currently no such classification for dogs and it is currently unknown whether dogs with CPS may have abnormal cerebrocortical activity in the absence of overt signs of neurological dysfunction, as described in humans.^14^ Electroencephalography may support the diagnosis of covert encephalopathy through generalized slowing of background rhythms^15^ interpreted alongside psychometric scores.^16^

Electroencephalography reflects the postsynaptic potentials of populations of pyramidal neurons in the cerebral cortex. These neurons are influenced by several biological factors that are particularly relevant in the setting of CPS and post-attenuation neurological dysfunction, including metabolic products such as ammonia^17^ and endogenous benzodiazepines,^18^ and therapeutic drugs such as the antiepileptic drug levetiracetam.^19^ In a previous case series, dogs with CPS and overt signs of HE all had abnormal findings on EEG.^20^ However, the prognostic value of EEG beyond the definition of overt neurological abnormalities in dogs with CPS is currently unknown. In addition, the syndrome of neurological dysfunction in dogs after CPS attenuation remains poorly understood and unpredictable.^4^

The objectives of this study were to prospectively monitor a cohort of dogs with CPS presenting with no overt signs of HE (1) to describe the spectrum of EEG manifestations consistent with covert HE before and after shunt attenuation, and (2) to assess the utility of specific EEG markers as predictors of post-attenuation outcomes. We hypothesized that perioperative EEG would reveal patterns consistent with covert HE in dogs with CPS, and that the presence EEG abnormalities would be associated with the development of post-attenuation neurological complications. To test this hypothesis, we performed prospectively pre- and postoperative EEG on a cohort of dogs undergoing CPS attenuation presenting with no overt signs of HE.

## Materials and methods

### Animals

Dogs with a presumptive diagnosis of CPS referred to the Small Animal Soft Tissue Surgery service for surgical evaluation between January 1st, 2022 and August 1st, 2024, were included in the study. Inclusion criteria were: (1) A diagnosis of either an intrahepatic or extrahepatic CPS confirmed by use of contrast computed tomography (CT), (2) No overt signs of HE at time of attenuation, which was defined as normal behavior and neurological examination, and (3) owner consent for enrollment in the study. Dogs were excluded if they had (1) abnormal neurological examination on admission, (2) additional vascular anomaly found on contrast CT, or (3) previously undergone CPS attenuation surgery.

### Clinical and clinicopathological data

Age, sex, breed, medications, and information relating to previous episodes of overt HE and seizures as recorded in the hospital medical record, primary veterinarian records, or reported by the caregivers, were obtained for all dogs. Each dog had a complete neurological examination at time of pre-attenuation EEG. Complete blood count, serum biochemistry, and venous ammonia concentration were measured at the time of initial EEG in a reference laboratory at the UC Davis Veterinary Medical Teaching Hospital. A second venous ammonia concentration was determined 24 hours after CPS attenuation.

The standard perioperative protocol for all CPS cases was to administer levetiracetam (60 mg/kg IV) at time of induction, and then 20-30 mg/kg IV or PO every 8 hours after attenuation. Attenuation of the CPS was done with ameroid ring for single extrahepatic CPS^21^ or stent-supported coil embolization for intrahepatic CPS,^22^ as previously described. For all cases of intrahepatic CPS, the caval and portal pressures were recorded intraoperatively via intravascular catheters before and after attenuation. Collection of liver biopsy at the time of surgery was performed at the surgeon’s discretion when attenuation of extrahepatic CPS was performed.

### Continuous EEG recordings in awake dogs

**Figure 1:**
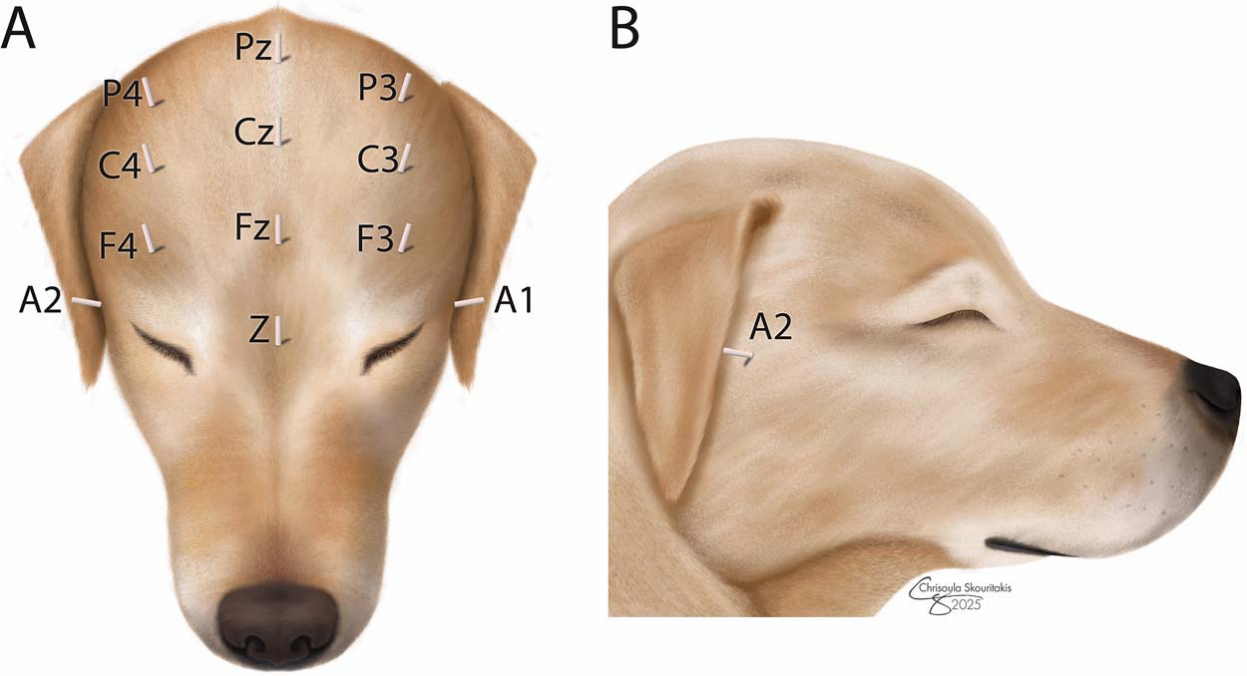
Schematic representation of electrode placement on a dorsal and right lateral view of a canine head. F = frontal; C = central; P = parietal; A = aural (placed just ventral to the zygomatic arch); Z = ground. Odd numbers = left side, even numbers = right side, z = midline.

Twelve subdermal 25-gauge wire EEG electrodes (Ives EEG Solutions, Newburyport, Massachusetts) were used as described^23^ (Figure 1). Electrodes were placed under general anesthesia after preoperative CT diagnostic imaging, and the dog’s head and electrodes were wrapped in bandage material to secure them in place.^23^ The electrodes were connected to an amplifier and recording unit, which were attached to a harness and worn by dogs weighing more than 10 kg; for smaller dogs, the equipment was placed in the cage with them. After anesthesia recovery, an EEG time-locked video camera was placed in front of the kennel to monitor the dog. Electroencephalography and video recording were started immediately and continued for 12-24 hours before CPS attenuation. The EEG recording unit was removed from the dog and the pre-attenuation recording was downloaded during the CPS attenuation procedure. The electrode harness and unit were re-attached during recovery from anesthesia, and EEG recording was continued 24-72 hours post-attenuation. Electroencephalography data were stored in an acquisition station for later analyses.

### Qualitative evaluation of initial EEG characteristics

The entirety of the EEG recordings of all dogs were examined using a 14-channel bipolar montage (sensitivity 7 μV/mm, high filter 70 Hz, low filter 1 Hz, and paper speed 30 mm/s) on an EEG recording platform (Cadwell Arc Alterna EEG, Kennewick, Washington) to report the background activity during sleep and awake states, presence of background slowing (defined as frequency of the background rhythm in the θ [4-8 Hz] or Δ range [0-4 Hz] in awake state^24^), epileptiform features, rhythmic or periodic patterns, and normal sleep transients (sleep spindle, K-complex, and vertex sharp wave). The term background activity was used to describe any sustained underlying EEG activity lasting more than 30 seconds. Normal awake background rhythm was defined as 10-25 Hz with variable amplitude below 50 μV, though higher amplitude could be observed in a young dog or dogs with poorly developed temporalis musculature.^25^

A designation of normal EEG was assigned to recordings with no epileptiform or encephalopathic features and normal background activity. The latter was defined based on sleep–wake stages as follows: episodes of arousal (background activity of high frequency 10-25 Hz and low amplitude < 30 μV), drowsiness, non-rapid eye movement sleep (background activity of low frequency 1-8 Hz and high amplitude > 30 μV) with normal sleep transients, including sleep spindles, K-complexes, vertex sharp waves, and rapid eye movement sleep (background activity of high frequency 10-25 Hz and low amplitude < 30 μV). A designation of abnormal EEG was assigned to recordings with any abnormality including: (1) epileptiform features including spikes (pointed transient, 20-70 ms, distinct from background, > 50 μV), spike-waves (spike followed by a slow wave, distinct from background activity), and polyspikes (sequence of 2 or more spikes),^24^ (2) rhythmic or periodic patterns, (3) absence of normal sleep architecture (defined as no distinct alteration in EEG background correlating with clinical arousal or sleep, and absence of any sleep transients), (4) burst activity (waveforms lasting ≥ 0.5 seconds and having at least 4 phases), (5) other encephalopathic features including diffuse slowing, lack of correlation between background activity and observation on time-locked videos, and triphasic waves.^26^

Based on combined EEG findings and clinical assessment, dogs were categorized into 2 groups. Dogs with no overt clinical signs of neurological dysfunction with a normal EEG were assigned to the “non-encephalopathic” group. Dogs with no overt clinical signs of neurological dysfunction with an abnormal EEG were assigned to the “covert encephalopathic” group.

### Quantitative EEG (qEEG)

Spectral analysis of digital EEG data was done using the EEG software Persyst V 12 (Persyst, Solana Beach, CA 92075, USA). The analysis was performed on the frequency spectrum 1-25 Hz using the transparietal derivation P3-P4 as described.^27^ The total duration of the EEG recording (T), regardless of its length, was divided by 4 to compartmentalize the EEG into 4 phases of equal durations. Four random start times within each phase were generated (1 for each phase) using a time generator (Random.org) to define the initiation point for each analysis. The EEG signal was visually inspected during the 80-second period after these times to ensure artifact-free intervals, characterized by absence of body movements, electrical interference, and movements of personnel or the patient visible in the transparietal derivation,^28^ or both. If an artifact was present, an additional random time was generated. This process was repeated until 4 artifact-free intervals of 80 seconds each were selected. Recordings deemed unsuitable for quantitative analysis due to a lost electrode at P3 or P4 were excluded from further analysis. Spectral analysis was performed over the selected EEG segment, then divided into 10 epochs of 8 seconds each. Specific parameters evaluated were (1) the median dominant frequency (MDF), corresponding to the mean power of each frequency weighted by the frequency rate, calculated using the formula $\frac{\sum_{i=1}^n\left({f}_i.{P}_i\right)}{\sum_{i=1}^n{P}_i}$, where ${f}_i$ corresponds to a specific frequency, ${P}_i$ the power associated with the frequency ${f}_i$, and *n* the number of frequencies evaluated; (2) the relative powers of the frequency bands δ [1-4 Hz], θ [4-8 Hz], α [8-13], and β [13-25 Hz], with each band inclusive of the lower boundary but not the upper boundary. ΔMDF was defined as the difference between pre- and post-attenuation MDF values (pre-attenuation − post-attenuation). Relative band power was calculated by normalizing the total power (1-25 Hz) for interindividual comparisons as previously described^29^; and (3) θ-to-β ratio, which is the ratio of the relative power of the frequency band θ over the frequency band β. The mean values for MDF, relative power, and ratio of all the epochs were collected and averaged for each patient. These parameters were selected based on evidence from human studies demonstrating improved assessment of mild HE by reducing interoperator variability and providing objective metrics correlated with mental status.^30^

### Statistical analysis

The statistical analyses were performed using Prism (Version 11; Graphpad, La Jolla, CA). The overall distribution of numerical data was assessed via graphic visualization and Shapiro–Wilk test. Data are presented as mean ± standard deviation for normally distributed variables or as median (range) for non-normally distributed variables. Paired pre- and post-attenuation values were compared using paired *t*-tests for normally distributed data or Wilcoxon signed-rank tests for non-normally distributed data. Correlations between continuous variables were assessed using Pearson or Spearman correlation tests, as appropriate. A *P*-value < .05 was considered statistically significant.

## Results

### Clinical features

A total of 28 dogs (21 males, 7 females) were prospectively enrolled in the study. Median age was 11.5 months (range 5-108). Mixed breed dogs were overrepresented (*n* = 6, 20%), followed by golden retriever (*n* = 4), Pug (*n* = 2), Labrador retriever (*n* = 2), Yorkshire terrier (*n* = 2), and Chihuahua (*n* = 2). Breeds with single cases included Siberian husky, bichon frisé, shih tzu, basset hound, French bulldog, Nova Scotia duck tolling retriever, border collie, papillon, Jack Russel terrier, and silky terrier.

All dogs had normal results on neurological examination and no episodes of overt encephalopathy or seizures during the 4 weeks that preceded surgery (*n* = 28). Observations consistent with neurological dysfunction before referral for CPS attenuation were documented in the medical records from referring veterinarians in 17 dogs (59%), and included change in mentation (*n* = 14), ataxia (*n* = 11), pacing (*n* = 9), and at least one reported generalized seizure (*n* = 3) before referral for CPS attenuation. Mean duration (±SD) from last sign of neurological dysfunction to presentation for attenuation of CPS was 9 (±4) weeks. Four (14%) dogs were receiving levetiracetam at time of referral (20-40 mg/kg PO every 8 hours). All 28 dogs were receiving lactulose, and 23 (80%) dogs were receiving an antibiotic, with the most common antibiotic utilized being amoxicillin (*n* = 19), followed by metronidazole (*n* = 4).

Fourteen dogs (50%) had attenuation of an extrahepatic CPS and 14 dogs (50%) had attenuation of an intrahepatic CPS. In dogs with extrahepatic CPS, the most common type was portocaval in 5 dogs (36%), followed by left gastrophrenic CPS in 4 dogs (29%), splenocaval in 3 dogs (21%), and gastrocaval in 2 dogs (14%). In dogs with intrahepatic CPS, there were 7 dogs (50%) with a right divisional CPS and 7 dogs (50%) with a left divisional CPS.

Three of the 28 dogs (11%) died or were euthanized before discharge because of immediate postoperative complications (portal hypertension, *n* = 1) or neurological deterioration and seizures (*n* = 2). The remaining 25 dogs had no immediate postoperative complications and survived to discharge.

All 28 dogs had diagnostic quality pre-attenuation EEG. Post-attenuation EEG was not obtained in 2 cases in the non-encephalopathic group due to equipment damage, resulting in diagnostic-quality post-attenuation EEGs being available for 26 dogs.

Twenty-four dogs (86%) were assigned to the “non-encephalopathic group,” as their pre-attenuation EEG was normal (Table 1 and Figure S1). Of these 24 dogs, 22 dogs had a normal post-attenuation EEG and survived until hospital discharge without postoperative complications. The remaining 2 dogs did not have a post-attenuation EEG performed; both had normal pre-attenuation EEG, had no postoperative complications in hospital, and survived to discharge.

**Table 1:** Clinical and electroencephalographic characteristics of the study groups.

	Dogs without encephalopathy (*n* = 24)	Dogs with covert encephalopathy (*n* = 4)
**Age median in months (range)**	10 (5-108)	84 (48-108)
**Sex**	19 Males 5 Females	2 Males 2 Females
**Historical seizures**	2/24	1/4
**Historical neurological signs**	14/24 (58%)	1/4 (25%)
**Receiving AED prior to initial EEG**	3/24	1/4
**Early onset seizures (<7 days post-attenuation)**	0/24 (0%)	2/4 (50%)
**In-hospital mortality (<7 days post-attenuation)**	0/24 (0%)	3/4 (75%)
**Ammonia pre-attenuation median in μg/dL (range)**	102.5 (34-401)	145.5 (56-275)
**Presence of paroxysmal discharges pre-attenuation**	0/24 (0%)	4/4 (100%)
**Presence of physiologic sleep transients pre-attenuation**	24/24 (100%)	2/4 (50%)
**ΔMDF (pre–post) median in Hz (range)**	0.14 (−0.65 to 0.79)	1.05 (0.34-1.1)

Abbreviations: AED = automated external defibrillator; EEG = electroencephalography; MDF = mean dominant frequency.

Four dogs (14%) were assigned to the “covert encephalopathic” group. These 4 dogs had normal pre-attenuation neurological exams, with abnormal pre- and post-attenuation EEG. Three dogs (75%) died or were euthanized before discharge. Only 1 dog out of 4 (dog 1) had historical signs of neurological dysfunction (ataxia, blindness, and obtunded), and generalized seizures (last seizure was 4 weeks before admission). Dogs 2, 3, and 4 had no history of seizures or signs of neurological dysfunction. Two of these dogs (dogs 1 and 2) had post-attenuation early-onset seizures and developed signs of encephalopathy, including altered state of consciousness, tetraparesis, and generalized ataxia. These signs of neurological dysfunction were refractory to medical treatment (including repeated boluses of 0.25 mg/kg midazolam IV or as constant rate infusion 0.2-1 mg/kg/h, levetiracetam 20-40 mg/kg up to every 6 hours, phenobarbital 3-4 mg/kg every 12 hours, and oral lactulose 130-530 mg/kg) which led to euthanasia of both dogs. One dog (dog 3) did not develop overt seizures, but this dog was severely obtunded postoperatively and deteriorated to being stuporous, the post-attenuation EEG was indicative of progressive encephalopathy with a predominant slow background 30-50 uV δ/θ rhythm and very few periods of EEG activity consistent with arousal (high frequency, low amplitude). This dog died after surgery due to complications associated with refractory portal hypertension. One dog (dog 4) with abnormal pre- and post-attenuation EEG did not develop seizures and survived to discharge.

The median age of dogs that developed early PAS was 78 months (range: 48-108) while that of dogs without seizures was 11 months (range: 8-108). Table 1 details the clinical and electroencephalographic characteristics of the studied groups.

Two out of 24 (8%) dogs with normal pre-attenuation EEG (non-encephalopathic group) developed late-onset seizures after discharge from the hospital (Figure S1). These 2 dogs both had historical neurological signs (ataxia, obtunded) but no seizures. Dog 5 developed focal facial seizures 1-month post-attenuation. Dog 6 developed generalized seizures at 15-month post-attenuation. Seizures in both dogs were well-controlled with levetiracetam (20-30 mg/kg PO q8h), with seizure frequency less than 1 every 3 months. Neither dog developed additional signs of neurological dysfunction and these dogs are still alive at time of manuscript writing.

### Electroencephalogram findings

#### E‌EG characteristics in the covert encephalopathy group (n = 4)

**Figure 2:**
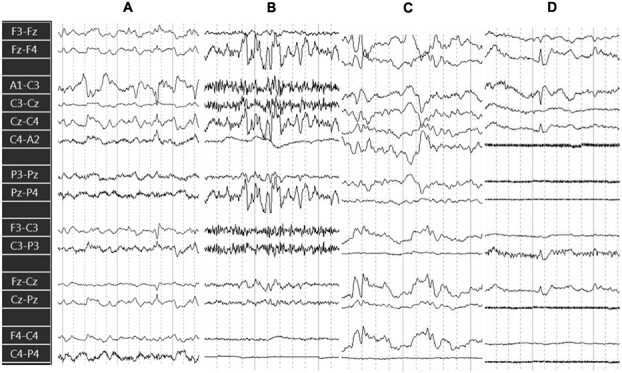
Composite of preattenuation EEG pattern examples from dogs with covert encephalopathy. Bipolar montage; recording parameters: sensitivity 7 μV/mm, high filter 70 Hz, low filter 1 Hz, and paper speed 30 mm/s. (A) Continuous high amplitude δ/θ activity with spikes (dog 1). (B) Burst of high amplitude δ/θ superimposed on fast activity (dog 2). (C) 90 μV polymorphic discharges 0.7-1 Hz (dog 3). (D) Isolated spike with background of α rhythm (dog 4). Abbreviation: EEG = electroencephalography.

**Figure 3:**
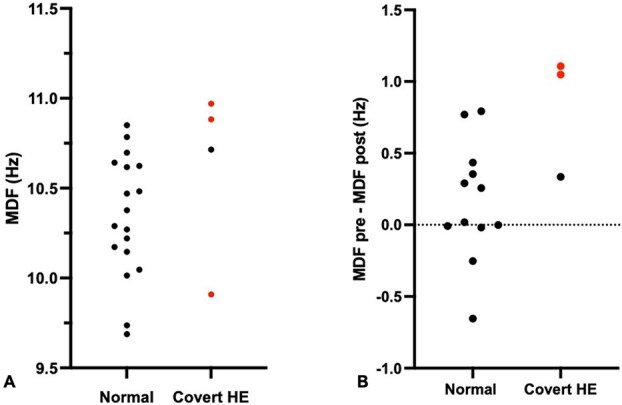
(A and B) Pre-attenuation MDF and difference in MDF (ΔMDF) before and after shunt attenuation. Red dots correspond to dogs that died (dogs 1, 2, and 3). Abbreviation: MDF = mean dominant frequency.

Abnormal qualitative features of pre- and post-attenuation EEG included (1) spikes, spike-waves, or polyspikes in all 4 dogs, (2) rhythmic or periodic patterns, observed in 3/4 dogs (dogs 1, 2, and 3) (Figure 2); (3) loss of distinct sleep and arousal stages in 2/4 dogs (dogs 1 and 2) with absence of normal sleep transients; and (4) burst activity in 1 dog (dog 2).

The prevalence of these abnormal features throughout the EEG recordings was notably different for the dog that did not develop PAS or other complications and survived to discharge (dog 4) compared to the non-survivors (dogs 1, 2, and 3). In dog 4, the EEG abnormalities were minimal and sporadic, considered < 1% of each total pre- and post-attenuation recording: a single spike was visualized in the pre-attenuation EEG with defined sleep/arousal stages and preservation of normal electrophysiologic sleep architecture and transients. Post-attenuation EEG showed rare polyspikes, with preservation of normal sleep architecture.

In contrast, dogs 1, 2, and 3 exhibited more prevalent pre-attenuation EEG abnormalities, in more than 50% of the total recording in each case. Post-attenuation EEG showed exacerbation of EEG abnormalities, with all 3 dogs developing nearly continuous abnormal rhythmic or periodic background patterns and frequent epileptiform features. None of these 3 dogs survived to discharge (Figure S1). Worsening of the EEG features of encephalopathy preceded the clinical onset of signs of neurological dysfunction in all cases by at least 24 hours.

Preoperative EEG abnormalities were present in the 2 dogs that developed early-onset PAS and all 3 dogs that died before discharge (including the 2 dogs with PAS), but were absent in all dogs that did not develop early-onset PAS.

#### Quantitative EEG

Quantitative EEG was successfully performed in 37/54 (69%) of recordings. The other 17 recordings (31%) were ineligible for quantitative analysis owing to insufficient EEG power and artifact impeding quantification in the biparietal derivation. This included 22 pre-attenuation EEGs (18 normal, 4 abnormal) and 15 EEG post-attenuation (12 normal, 3 abnormal). Therefore, 15 dogs had both pre- and post-attenuation qEEG available, including 3 dogs with covert encephalopathy, and 12 non-encephalopathic dogs. Before attenuation, the median (range) MDF was 10.3 (9.7-10.9) in dogs without encephalopathy and 10.8 (9.9-11.0) in those with covert encephalopathy (Figure 3A). After attenuation, the median MDF was 10.2 (9.7-10.7) in dogs without encephalopathy and 9.9 (9.8-10.4) in those with covert encephalopathy. Mean dominant frequency showed significant changes from pre-attenuation to post-attenuation (median differences [range] = 0.29 [−0.65 to 1.1]; *P* = .03; *n* = 15). ΔMDF was 1.05 and 1.1 in 2 dogs that died, respectively, while the median ΔMDF for survivors was 0.26 (−0.65 to 0.79; *n* = 13). Pre- and post-attenuation differences were not observed in the relative power of the δ (*P* = .76), θ (*P* = .64), α (*P* = .890), nor β (*P* = .135) frequency bands over the parietal regions (*n* = 15). Similarly, the θ/β ratio was not different pre- vs post-attenuation (*P* = .1205; *n* = 15). There was a moderate correlation between patient age and MDF (Spearman *r* [95% CI] *r* = 0.57 [0.18-0.80] *P* = .0056; *n* = 22), but not between serum ammonia concentration and MDF (−0.02 [−0.45 to 0.42] *P* = .93; *n* = 22).

### Ancillary diagnostic results

#### Ammonia

The median pre-attenuation serum ammonia concentration was 108.5 μg/dL (range 34-401) (laboratory reference interval 0-59 μg/dL), which was elevated in 25/28 dogs, including 3/4 (75%) dogs with covert encephalopathy and 22/24 (92%) dogs without encephalopathy. Post-attenuation serum ammonia concentrations were elevated in 11/24 dogs (median serum ammonia 48.5 μg/dL [4-591]). Three dogs that did not have elevated pre-attenuation serum ammonia concentrations had greater post-attenuation concentrations (1 dog with covert encephalopathy and 2 dogs without encephalopathy). Median ammonia concentration was 102.5 μg/dL (34-401) in non-encephalopathic dogs and 145.5 μg/dL (56-275) in dogs with covert encephalopathy.

#### Portal pressure

For intrahepatic CPS (*n* = 14), the caval and portal pressures before attenuation had a median (range) of 5 (−2 to 16) mmHg and 7 (0-19) mmHg, respectively, and after attenuation 4 (−2 to 14) mmHg and 10 (3-28) mmHg, respectively. There was a significant difference between pre-attenuation and post-attenuation portal pressure (median difference [range] 3.000 [−1 to 9], *P* < .0001), but not for the caval pressure (median difference 0 [−6 to 2]; *P* = .08). In this cohort, no dogs with intrahepatic CPS developed early onset seizures; however, 1 dog with intrahepatic CPS developed late onset-seizures (dog 5).

### Histopathology

**Figure 4:**
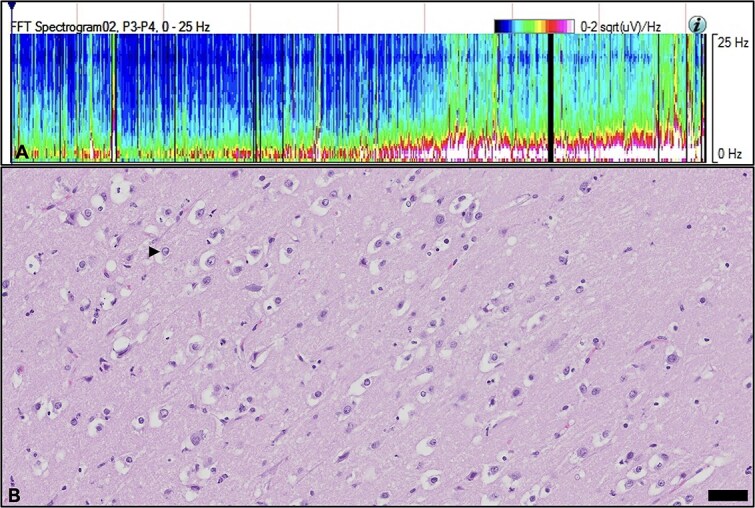
Quantitative EEG and brain histopathology in a dog (dog 2) with covert encephalopathy that developed seizures 25 hours after attenuation of an extrahepatic shunt. (A) Postattenuation power spectral density of EEG channels P3-P4. There is a progressive increase in power within the low-frequency bands δ (0-4 Hz) and θ (4-8 Hz) with concurrent reduction in β activity (13-25 Hz). This spectral shift suggests a gradual slowing of cortical activity. The pattern is consistent with progressive encephalopathy after attenuation of the shunt and before development of seizures. (B) Histopathology of the cerebral cortex in the same dog after euthanasia due to the development of refractory seizures. Figure shows cerebral edema and Alzheimer type II astrocyte (arrow). Bar is 50 μm. Abbreviation: EEG = electroencephalography.

Histopathology of liver biopsy collected in surgery was available for 12 dogs (8/24 non-encephalopathic and 4/4 covert encephalopathic dogs). The most common finding in non-encephalopathic dogs was mild lobular atrophy (8/8 dogs), while mild portal vein fibrosis was reported in 1/8 dogs. All covert encephalopathic dogs (*n* = 4) had moderate portal vein fibrosis. Histopathology of the brain was available for 1 dog with covert encephalopathy EEG that developed PAS (Figure 4), which revealed cerebral edema, gliosis with Alzheimer type II astrocytes, and deep cortical laminar necrosis.

## Discussion

Neurologic morbidity is a reported complication in dogs after CPS attenuation.^4^^,^^5^^,^^8^ This study indicates that EEG evaluation allows for recognition of abnormal cerebral activity pre-attenuation, even in the absence of overt clinical signs at time of examination, and that these dogs have apparently higher risk of experiencing poorer outcome including seizures, change in mentation, and death in the short term.

The diagnosis of HE in dogs with CPS traditionally relies on the clinical examination in conjunction with serum biomarkers such as elevation in ammonia or bile acids, or both, and does not routinely include the use of EEG. In this study, EEG provided evidence of abnormal cerebral activity in dogs with CPS without overt signs of HE. In particular, ambulatory EEG allowed evaluation of background activity, normal and abnormal transients over a prolonged period throughout the sleep–wake cycle. Pre-attenuation EEG in dogs that subsequently developed PAS revealed abnormal background rhythms in both wakefulness and sleep with loss of normal sleep cycle patterns and sleep related graphoelements.

Abnormal structure of nocturnal sleep and alternations of MDF during sleep is an early marker of cerebral dysfunction in humans with cirrhosis.^31^ For these patients, EEG has prognostic value regarding the occurrence of both HE and liver disease-related death, and the assessment of MDF is used to score end-stage disease. Although diffuse background slowing could potentially complicate recognition of distinct sleep stages, the use of time-locked video in this study facilitated EEG interpretation by demonstrating a continuum of abnormal background activity between the awake EEG and the sleep EEG during apparent sleep. In children with acute liver failure, EEG helps quantify and categorize the grade of HE.^32^ One of the earliest electroencephalographic changes in HE preceding background slowing is an increase in fast (β) activity.^30^^,^^33^^,^^34^ This might explain findings in some dogs with covert encephalopathy, where relatively high baseline MDF were observed before overt background slowing in the post-attenuation setting. In the present study, MDF decreased after attenuation, suggesting that relative change from baseline, rather than the absolute value, might provide a more meaningful neurophysiologic evaluation. In patients with covert encephalopathy, MDF values decline with progression to overt encephalopathy, correlating with worse psychometric scores and more advanced liver disease.^28^ In addition, lower MDF values are significantly associated with reduced survival.^35^

The presence of graphoelements of sleep was also relevant in the evaluation dogs with CPS. In people with HE, the lack of vertex sharp-waves, sleep spindles, and K-complexes are associated with unfavorable outcome.^36^ Similarly in this study, dogs that developed PAS lacked physiologic sleep transients in their EEGs, suggesting that the loss of physiological graphoelements of sleep is an indicator of susceptibility to neurological dysfunction associated with HE and carries prognostic significance. While sleep disruption is reported in dogs hospitalized in ICU,^37^ all dogs were hospitalized in general ward before the shunt attenuation procedure and showed apparent sleep behavior on time-locked video.

Epileptiform features and rhythmic or periodic discharges helped characterize pre-attenuation EEG associated with the development of neurological dysfunction in the post-attenuation setting. In dogs with covert encephalopathy, frequent periodic discharges superimposed on abnormal background activity were observed before the development of overt seizures. Only one dog with PAS had documented seizures before referral, and the last witnessed seizure was 4 weeks before admission for CPS attenuation. These findings suggest that certain patterns of EEG are associated with the development of overt HE and greater likelihood of seizures post-CPS attenuation, particularly when observed in the absence of normal background rhythm and loss of sleep architecture and transients. In people undergoing liver transplantation, nonsurvivors show more frequent abnormal paroxysmal discharges on EEG than survivors.^38^ This mirrors observations in the present study, where preoperative EEG abnormalities were associated with a higher incidence of postoperative mortality. It is worth noting that one dog had an isolated epileptiform feature with an overall normal background activity, and this pattern remained stable in the postoperative setting, with the patient not experiencing adverse events. This observation supports HE as a continuum of clinical and electrophysiological severity. Triphasic waves are reported in dogs with HE,^20^ and their absence in this study might reflect variability in EEG manifestations of HE, or might suggest that acute hyperammonemia, covert encephalopathy, and PAS involve distinct pathophysiological mechanisms. While triphasic waves are seen with advanced encephalopathy,^11^^,^^34^ they are not pathognomonic for HE; more recently the terminology “generalized periodic discharges with triphasic morphology”^39^ reflects their classification as a subtype of generalized periodic discharges rather than a disease-specific finding.^40^^,^^41^

Various pathophysiological processes can affect cerebral physiology, leading to qEEG changes such as power of the different frequency bands and dominant frequency, that might not be immediately apparent through visual EEG examination.^42^^,^^43^ Quantitative EEG has been used in people for assessing HE in acute and chronic settings.^44^ In dogs with covert encephalopathy undergoing CPS attenuation, MDF appears as a relevant parameter for monitoring cortical activity over time, as a substantial decline in MDF was observed in dogs with poor outcome, and it is an objective value that can be monitored serially.

Two dogs with normal pre-attenuation EEG developed seizures more than 1 month after discharge from the hospital, without other neurological signs. The clinical characteristics of these late-onset seizures appear more benign as these dogs survived with relatively infrequent seizures. Their etiopathophysiology is uncertain, and whether later-onset seizures reflect a shared pathology or independent entities remains unclear. The parameters described above were valuable to discriminate early-onset but not late-onset cases, indicating that a normal pre-attenuation EEG does not rule out the possibility seizures at a later stage.

In people with liver diseases, risk factors for the development of covert HE include older age,^9^^,^^45^ degree of liver damage,^13^^,^^15^^,^^45–48^ and previous clinically apparent HE episodes.^15^ Age has previously been proposed as a potential risk factor for PAS in dogs,^8^ possibly due to prolonged exposure to neurotoxic compounds capable of inducing cerebral pathology. In our study, although dogs with PAS tended to be older, not all older dogs showed EEG abnormalities nor developed PAS, and the occurrence of historical HE episodes was common in dogs with normal EEG (58%). In a previous study,^8^ dogs exhibiting encephalopathic signs immediately before CPS attenuation were at greater risk for PAS, suggesting that presurgical alterations in the central nervous system might contribute to the emergence of new neurological dysfunctions. In our study, one dog with PAS had brain histopathology performed confirming brain edema and neuronal necrosis. Astrocytic swelling secondary to nitrogenous metabolite accumulation is suspected to be a contributor to worsening neurological signs with HE.^49^^,^^50^ In humans, serum ammonia concentrations do not consistently correlate with HE severity,^51^ likely due to altered cerebral ammonia metabolism in chronic disease.^52^ Chronic low-grade hyperammonemia might deplete astrocytic glutamate, promoting cortical inhibition and leading to covert HE through mechanisms involving cerebral edema.^53^

This study has several limitations. First, only a limited number of dogs developed post-attenuation complications, which restrict the extrapolation of these observations. Second, spectral analysis of ambulatory EEG in dogs is constrained by movement artifacts, which can interfere with cortical bioelectrical signals. These artifacts might overlap with the δ frequency range, causing a spurious increase in δ power and complicating the detection of subtle changes in lower frequency bands. However, the diagnostic value of ambulatory EEG monitoring outweighed this limitation, as the recognition of abnormal background through the sleep–wake cycle over a prolonged time period was a prognostic factor for the development of neurological signs and for short-term mortality. Electroencephalography is subject to considerable variability due to both intra- and interindividual factors, including biological (eg, skull and temporalis muscle thickness, pharmacological influences), technical (eg, electrode placement, impedance, and derivation), and artifactual issues. In this study, only the transparietal derivation was assessed quantitatively. Numerical values associated with qEEG might not be translatable to another laboratory where equipment, techniques, and recording settings might result in significant differences.

This study does not attribute EEG changes specifically to shunt attenuation, as multiple uncontrolled variables occurred in the same timeframe, including general anesthesia, hepatic manipulation, fluctuations in ammonia, and alterations in systemic and portal pressures, and postanesthetic signs of neurological dysfunction might occur in dogs with unoperated CPS after non–shunt-related surgery.^54^

### Conclusion

Electroencephalography analysis during the perioperative period enabled identification of covert encephalopathy in a subset of dogs with CPS. The presence of specific EEG abnormalities could aid in the identification of dog with CPS that are more likely to develop post-attenuation complications. Dogs with no abnormalities detected on pre-attenuation EEG appear to have positive outcomes during their hospitalization. Identification of high-risk cases might enable implementation of perioperative targeted therapeutic strategies to decrease neurological-associated morbidity and death and might allow for further investigation of pathophysiology in defined clinical cases.

## Supplementary Material

aalaf051_Supplemental_Files

## Off-label antimicrobial declaration

The authors declare no off-label use of antimicrobials.

## Institutional animal care and use committee or other approval declaration

Approved by the University of California Davis Institutional Animal Care and Use Committee (Protocol number: 22652).

## Abbreviations

CPS: congenital portosystemic shunts
CT: computed tomography
qEEG: quantitative electroencephalography
HE: hepatic encephalopathy
MDF: mean dominant frequency
PAS: post-attenuation seizures
