# Supplementary material for: Perioperative assessment of electroencephalography in dogs with congenital portosystemic shunts
Source: J Vet Intern Med. 2026 Jan 21;40(1):aalaf051. doi: 10.1093/jvimsj/aalaf051 (PMC12881966; doi:10.1093/jvimsj/aalaf051)
Supplement: aalaf051_Supplemental_Files [file aalaf051_supplemental_files.zip › Supplemental_Figure_caption_aalaf051.docx]

**Supplemental Figure 1:** Post-attenuation outcome of dogs and individual EEG observations

Abnormal EEG findings included: a) Spikes, b) Epileptiform features other than spikes, c) Abnormal sleep architecture, d) Rhythmic periodic pattern.
