# Supplementary figures and images for: Perioperative assessment of electroencephalography in dogs with congenital portosystemic shunts
Source: J Vet Intern Med. 2026 Jan 21;40(1):aalaf051. doi: 10.1093/jvimsj/aalaf051 (PMC12881966; doi:10.1093/jvimsj/aalaf051)

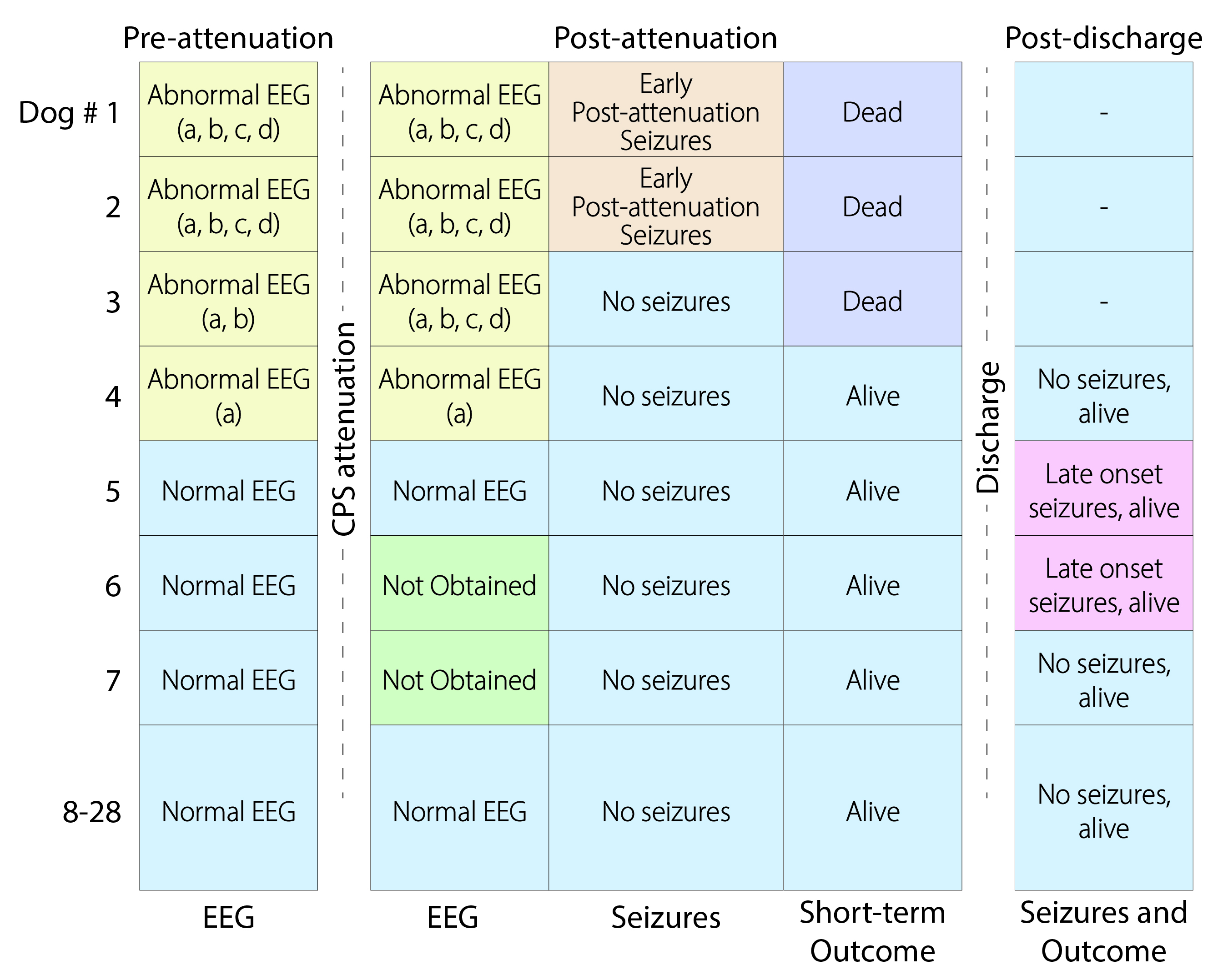

Supplement: aalaf051_Supplemental_Files [file aalaf051_supplemental_files.zip › supplemental_fig_1_aalaf051.jpg]
